# Association between displacement and thickness of macula after vitrectomy in eyes with epiretinal membrane

**DOI:** 10.1038/s41598-020-70197-6

**Published:** 2020-08-06

**Authors:** Ayana Momota, Takeshi Iwase, Tomohiko Akahori, Kensuke Goto, Kentaro Yamamoto, Eimei Ra, Hiroko Terasaki

**Affiliations:** 1 Department of Ophthalmology, Nagoya University Graduate School of Medicine, 65 Tsurumai-cho, Showa-ku, Nagoya, 466-8560 Japan; 2 Department of Ophthalmology, Akita University Graduate School of Medicine, 1-1-1 Hondou, Akita, 010-0041 Japan

**Keywords:** Outcomes research, Medical imaging

## Abstract

The purpose of this cross-sectional retrospective study was to determine the relationship between the retinal displacements and the retinal thickness in eyes with epiretinal membrane (ERM) after vitrectomy with internal limiting membrane (ILM) peeling. To accomplish this, we measured the retinal thickness using optical coherence tomography (OCT) and the retinal displacement using OCT angiography to obtain 3 mm × 3 mm en face images before, and 2, 4, and 8 weeks following the surgery from 20 eyes of 20 patients. The distance between the retinal vessel bifurcations and the fovea was significantly displaced centrifugally and asymmetrically in the 4 quadrants postoperatively (*P* < 0.001). The foveal avascular zone (FAZ) was significantly enlarged, and the central foveal thickness (CFT) and the inner nuclear layer (INL) thickness were significantly thinner postoperatively. The displacements were significantly correlated with the changes in the FAZ area (r = 0.717, *P* < 0.001), the CFT (r = − 0.702, *P* < 0.001), and the INL thickness (r = − 0.702, *P* < 0.001). In conclusion, the distance between the retinal bifurcations and the fovea was asymmetrically expanded after the surgery and was significantly correlated with the morphological changes. These results indicate that a horizontal macular contraction is correlated with vertical retinal contraction in the eyes with an ERM.

## Introduction

An idiopathic epiretinal membrane (ERM) is a relatively common retinochoroidal disorder in older individuals^1,2^, and it can cause an inner retinal displacement of the macula area and a disruption of the foveal structure^3^. These alterations cause traction on the retina in the macular area which can lead to visual dysfunction and metamorphopsia. Vitrectomy and removal of the ERM with or without internal limiting membrane (ILM) peeling is used to release the traction and is effective in improving the visual dysfunction, metamorphopsia, and visual acuity^4–6^. Thus, it is important to know how the retina in the macular area is displaced in more detail after the surgery because the displacement will affect the visual function.

Optical coherence tomography (OCT) enables clinicians to obtain information of the microstructures of the fovea, and earlier studies have reported that the retinal layer at the fovea is thickened and the outer retinal layers are disrupted in eyes with an ERM^7–9^. The integrity of the outer retinal layers is important for vision in retinochoroidal disorders, and their alterations have been evaluated^10–12^. However, there have been only a few reports on the changes of the inner retinal layers in eyes with ERM after surgery^13^. This is important because the integrity of the inner retina is a major determinant of the visual dysfunctions in eyes with an ERM.

OCT angiography (OCTA) is a noninvasive and safe technique that can obtain images of the microvasculature of the retina and the choroid. OCTA has enabled clinicians to investigate the retinal vasculature in situ repeatedly in short intervals^14,15^. The displacements of the inner retina can be evaluated using the superficial OCTA *en face* images of the retina before and after surgery^16^. Thus, OCTA is a suitable method for evaluating whether there is a displacement of the inner retina postoperatively. Akahori et al. evaluated the displacement of the inner retina after surgery in eyes with a macular hole^16^. However, there has not been a study that investigated the displacement of the retina after vitrectomy that removed an ERM.

Thus, the purpose of this study was to determine the relationship between the displacements of the inner retina and the retinal thicknesses in eyes with an ERM after vitrectomy with ILM peeling. To accomplish this, the superficial foveal avascular zone (FAZ) and the displacement of the retina were measured in the OCTA images, and the retinal thickness including the central foveal thickness (CFT) was measured in the spectral-domain OCT (SD-OCT) B scan images of eyes with an ERM before and after its removal. In addition, the relationships between the values determined by OCTA and SD-OCT were evaluated after vitrectomy with ILM peeling for the removal of an ERM.

## Methods

### Subjects

We reviewed the medical records of all patients with a unilateral, idiopathic ERM who had undergone vitrectomy with ILM peeling at the Nagoya University Hospital from December 2016 to March 2019. All of the patients had undergone comprehensive ophthalmic examinations. Additionally, OCTA and SD-OCT (Spectralis^®^, Heidelberg Engineering, Heidelberg, Germany) images were obtained before, and 2, 4, and 8 weeks following the surgery. The size and the location of the ERM and ILM peeling were determined from the videos recorded during the surgery. Eyes with severe cataract, macular diseases, history of other ocular diseases, and high myopia of > − 5 diopters (D) were excluded.

### Measurements using optical coherence tomographic images

**Figure 1:**
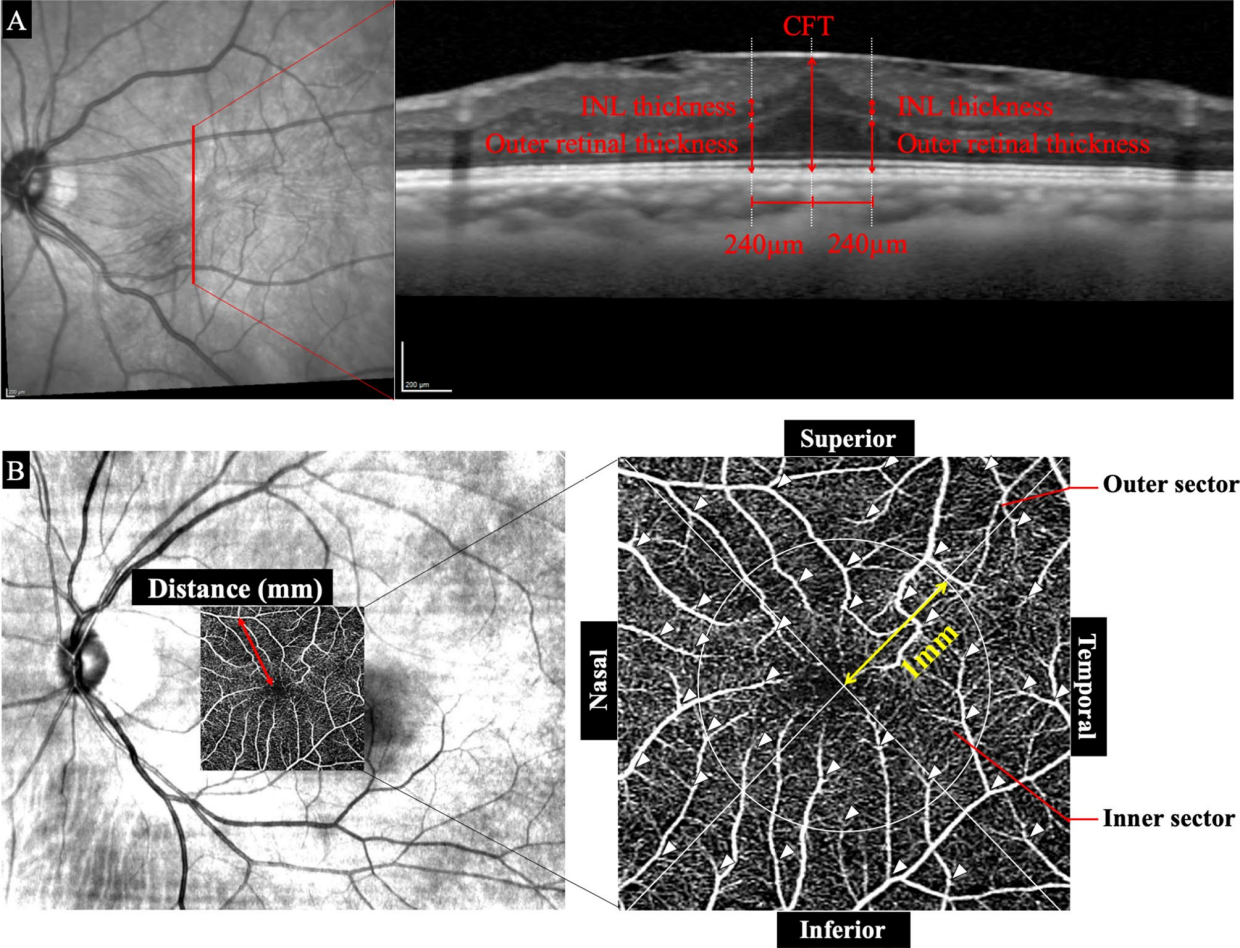
Preoperative fundus photographs and optical coherence tomographic (OCT) images of a 67-year-old man with an idiopathic epiretinal membrane (ERM). (**A**) Spectral domain OCT image (Spectralis). (**B**) Superficial foveal retinal capillary plexuses observed in OCT angiography (OCTA) image. The central foveal thickness (CFT) was measured as the distance between the surface of the ILM and the outer border of the retinal pigment epithelium at the center of the fovea. The inner nuclear layer (INL) thickness and the outer retinal layer (ONL) thickness were measured at 240 µm away from the fovea. The preoperative fundus photograph and a superimposed OCTA image at the superficial retinal capillary network layer. The retinal vasculature is shown by the white lines. The OCTA images are divided into 4 sectors by drawing a line from the retinal vessel bifurcations to the fovea. Then a line was drawn perpendicular to the original line. This divided the retina into the temporal, superior, nasal, and inferior quadrants. In addition, each quadrant was subdivided into an inner region and an outer region. The inner sector was defined as the region within a distance of 1 mm from the fovea and outer sector was outside the 1 mm region.

We analyzed all of the horizontal and vertical cross-sectional Spectralis^®^ OCT images recorded at each visit after the surgery. The retinal layer thicknesses were measured on the same selected central foveal location during the follow-up period with the embedded caliper measurement tool in the OCT. The CFT was defined as the thickness from the surface of the ILM to the outer border of the RPE at the central fovea (Fig. 1A). The thickness of the inner and outer retinal layers was measured at the superior, temporal, inferior, and nasal quadrants at 240 µm away from the center of the fovea in the vertical and horizontal scan images. The inner layer (INL) thickness and outer retinal layer (ORL) thickness (outer nuclear layer + outer plexiform layer) of these 4 quadrants were measured with the caliper function.

### Optical coherence tomography angiography (OCTA)

**Figure 2:**
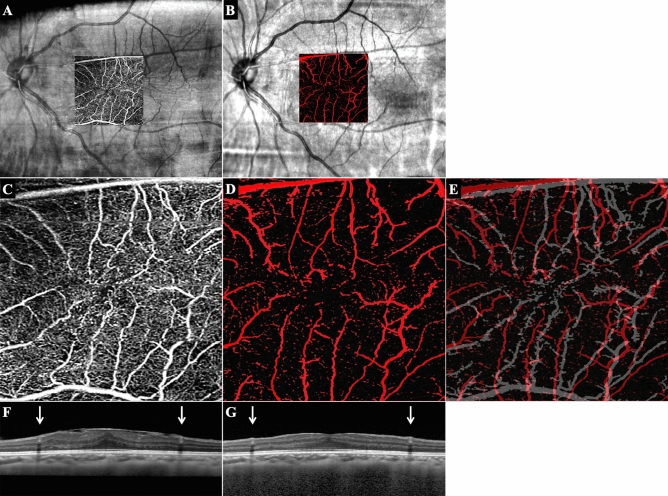
Representative pre- and postoperative fundus and OCT and OCTA images. Preoperative fundus photograph and a superimposed OCTA image of the superficial retinal capillary network of a 72-year-old woman with an ERM (**A**). The retinal vasculature is shown by the white lines. The postoperative fundus photograph with an OCTA image at Week 2 after the vitrectomy (**B**). The retinal vasculature is shown as red lines. The magnified OCTA preoperative image (white, **C**) and postoperative image (red, **D**) are superimposed in **E**. The images of the pre- and postoperative images were adjusted by matching retinal vessels around the optic disc. Pre- (**F**) and postoperative (**G**) SD-OCT images. The distance between the arrows was longer after the surgery.

We obtained the 3 × 3 mm *en face* images of the microvasculature in the macular region using the Angioplex (Zeiss Meditec. Inc, Germany) OCTA device^16^. The images of the superficial capillary network layer were used to measure the displacements of the retina and the area of the foveal avascular zone (FAZ; Fig. 2).

All of the images were reviewed by the two graders (AM and TI) and low quality images were excluded from the analyses. The two graders measured the vascular displacements independently and were masked of the other clinical findings.

### Measurements of displacement of retina using OCTA images

The displacement of retina was measured using the OCTA images as described in detail^16^. We adjusted the changes between the pre- and postoperative OCTA images by matching the retinal vasculature around the optic disc (Fig. 1B). The displacements of the retina were made relative to the fovea. To do this, we measured the distance between the fovea and an easily identifiable retinal vessels bifurcation pre- and postoperatively.

We divided the OCTA *en face* images into 4 quadrants as the superior, temporal, inferior, and nasal quadrants (Fig. 1B). Additionally, we divided each quadrant into an inner and outer region. The inner region was defined as the area within 1 mm from the fovea and the outer region was > 1 mm peripheral to the fovea. The average of the changes in the distance in each quadrant and region was determined.

### Surgical techniques

A standard 25-gauge (G) three-port pars plana vitrectomy (PPV) was performed by one surgeon (TI) using the Constellation system (Alcon Laboratories, Inc., Fort Worth, TX, USA). After core vitrectomy, the ERM and ILM were peeled circumferentially from the retina with ILM-peeling forceps in all cases. Finally, we performed a peripheral vitrectomy with shaving and carefully inspection of the periphery over 360 degrees.

Cataract surgery and implanted a foldable acrylic IOL into the capsular bag were performed on all 19 phakic eyes.

### Statistical analyses

One-way analysis of variance (ANOVA) was used to determine the significance of the correlations between the displacements in the four quadrants and the time of the surgery. Pearson’s correlation coefficient tests were used to determine the significance of the associations between the changes in the displacement of each bifurcation and other variables. All statistical analyses were performed using the Statistical Package for Social Sciences for Windows 21.0 (SPSS Inc, Chicago, IL).

### Ethics statement

The Ethics Committee of Nagoya University Hospital approved the procedures used in this cross-sectional retrospective study. The procedures conformed to the tenets of the Declaration of Helsinki. All of the patients signed an informed consent form before the surgery.

## Results

### Demographics of patient

Forty-one eyes of 41 patients with an ERM underwent vitrectomy with ILM peeling and SD-OCT and OCTA examinations between December 2017 and March 2019. Twenty-one eyes were excluded: 8 for a pseudo macular hole, 3 for macular degeneration, 1 for macular edema, 3 for severe cataract that prevented obtaining high quality OCTA images, and 6 moved to another hospital immediately after the surgery that did not have sufficient postoperative examinations. In the end, 20 eyes of 20 patients (mean age, 70.6 ± 6.6 years) were studied. The demographics of the patients and the surgical procedures are shown in Table 1.

**Table 1:** Clinical characteristics of subjects.

Characteristics	n = 20
Age (year)	70.6 ± 6.6
Gender (male/female)	5/15
Affected eye (right/left )	10/10
Axial length (mm)	23.7 ± 1.1
Preoperative BCVA (logMAR)	0.22 ± 0.21
Postoperative BCVA (logMAR)	0.09 ± 0.14
Preoperative FAZ size (mm^2^)	0.065 ± 0.03
PPV + PEA + IOL/PPV (eyes)	19/1
ILM peeling size (DD)	4.78 ± 0.44

Values are presented as numbers or as means ± standard deviations.

*BCVA* best-corrected visual acuity, *logMAR* logarithm of minimum angle of resolution, *FAZ* foveal avascular zone, *PPV* pars palana vitrectomy, *PEA* phacoemulsification and aspiration, *IOL* intraocular lens, *DD* disc diameter.

The mean postoperative best-corrected visual acuity (BCVA) was significantly improved (*P* < 0.001). The mean size of the ILM peeled area was 4.78 ± 0.44 disc diameters (DDs).

### Changes in distance between retinal bifurcations and fovea

The mean number of retinal bifurcations that was identified was 71.0 ± 16.1/eye. The repeatability of the changes in the distance between the fovea and retinal vessel bifurcations between the graders was excellent with an ICC of 0.99.

**Figure 3:**
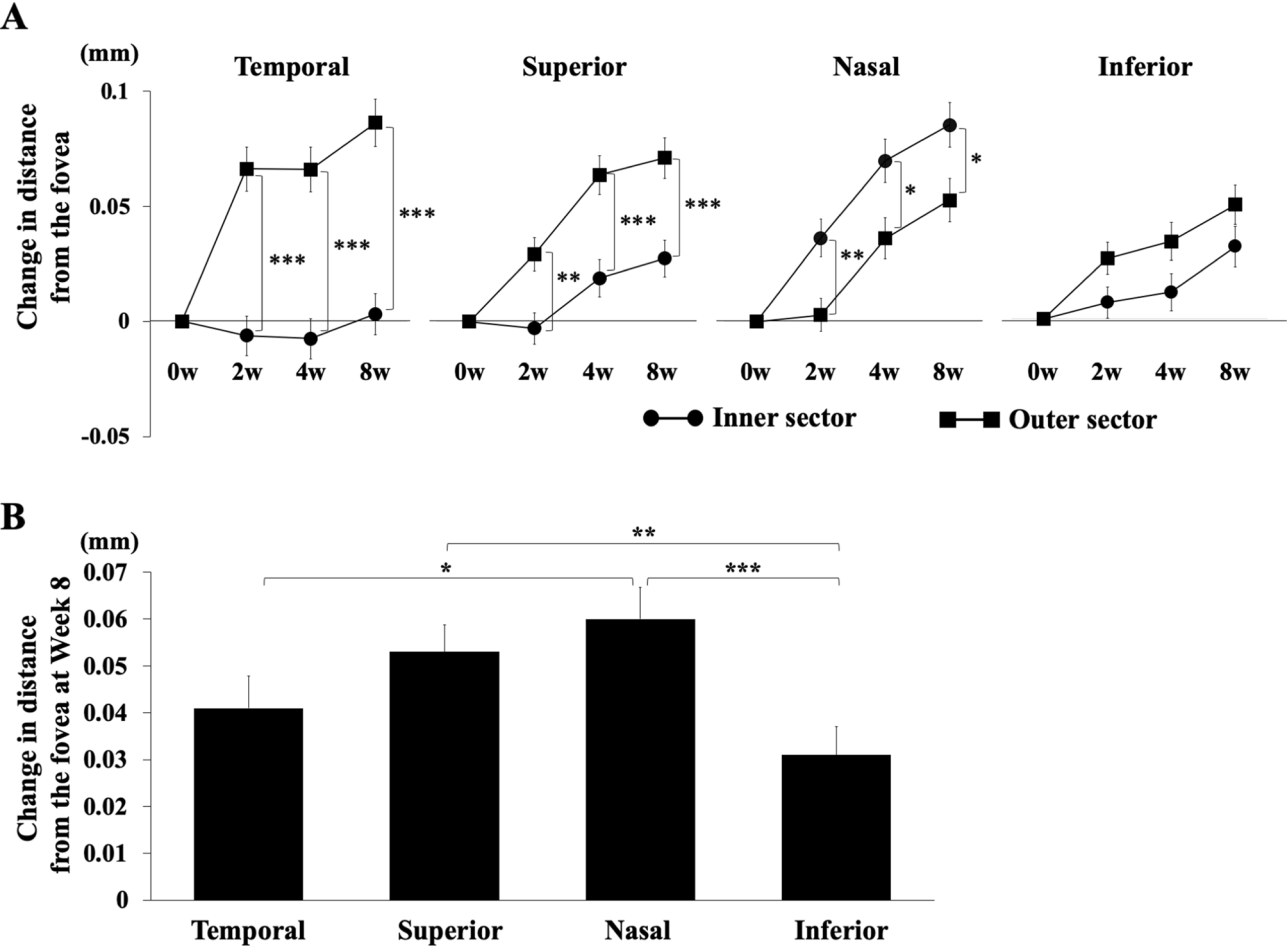
Displacements in the four quadrants after surgery and comparisons among the quadrants. The distance from the fovea and retinal vessel bifurcations was significantly shorter in all of the quadrants throughout the postoperative period (*P* < 0.001) (**A**). The outer sector was significantly displaced in the temporal and the superior quadrants during the follow-up period, but the inner sector was significantly displaced only in the nasal quadrant during the follow-up period. Comparisons of the changes in the distances in the 4 quadrants at Week 8 (**B**). The distance in the nasal quadrant was significantly greater than that in the temporal and inferior quadrants. The distances in the superior quadrant were significantly greater than that in the inferior quadrant.

The distance between the fovea and retinal vessel bifurcations was significantly greater after the vitrectomy than before in all four quadrants (*P* < 0.001; Table 2), and the distance increased during the follow-up period (*P* < 0.001; Fig. 3A, Table 2). The outer sectors were significantly displaced in the temporal and the superior quadrants during the follow-up period, but the inner sector was significantly displaced only in the nasal quadrant during the follow-up period.

**Table 2:** Change in distance between bifurcations and the fovea.

Quadrant	Week 2	Week 4	Week 8	*P* value
**Temporal (mm)**				
All	0.031 ± 0.126	0.029 ± 0.129	0.045 ± 0.137	< 0.001
Inner	− 0.006 ± 0.111	− 0.008 ± 0.116	0.003 ± 0.120	0.719
Outer	0.066 ± 0.130	0.066 ± 0.131	0.086 ± 0.138	< 0.001
**Superior (mm)**				
All	0.020 ± 0.095	0.051 ± 0.112	0.066 ± 0.115	< 0.001
Inner	− 0.003 ± 0.086	0.019 ± 0.104	0.028 ± 0.102	0.002
Outer	0.036 ± 0.100	0.064 ± 0.116	0.072 ± 0.122	< 0.001
**Nasal (mm)**				
All	0.018 ± 0.092	0.051 ± 0.111	0.066 ± 0.115	< 0.001
Inner	0.036 ± 0.093	0.069 ± 0.106	0.085 ± 0.109	< 0.001
Outer	0.003 ± 0.090	0.036 ± 0.112	0.053 ± 0.118	< 0.001
**Inferior (mm)**				
All	0.013 ± 0.093	0.015 ± 0.105	0.030 ± 0.118	< 0.001
Inner	0.007 ± 0.100	0.012 ± 0.118	0.031 ± 0.129	0.010
Outer	0.026 ± 0.082	0.034 ± 0.098	0.050 ± 0.100	< 0.001

Values are presented as means ± standard deviations.

The changes in the distance between the fovea and retinal vessel bifurcations at Week 8 was selected to compare the alterations in the 4 quadrants. The change in the distance in the nasal quadrant was significantly greater than that in the inferior (*P* < 0.001) and temporal (*P* = 0.036) quadrants, and it was significantly greater in the superior quadrant than the inferior quadrant (*P* = 0.005; Fig. 3B).

### Changes in foveal avascular zone (FAZ) area

The mean preoperative FAZ area was 0.06 ± 0.03 mm^2^ in eyes with ERM, and it was significantly smaller than that of the fellow eye at 0.38 ± 0.07 mm^2^ (*P* < 0.001). The mean postoperative FAZ was 0.07 ± 0.03 mm^2^ at 2 weeks, 0.08 ± 0.03 mm^2^ at 4 weeks, and 0.09 ± 0.03 mm^2^ at 8 weeks after surgery. The increase in the area during the postoperative period was significant (*P* < 0.001). Of the 20 eyes, the area of FAZ in 18 eyes became larger after surgery.

### Changes in retinal layer thickness

The mean preoperative CFT area was 406.3 ± 79.9 µm in eyes with an ERM which was significantly thicker than that of the fellow eye at 183.8 ± 16.7 µm (*P* < 0.001). The mean postoperative CFT was significantly thinner than the baseline thickness during the entire follow-up period (*P* = 0.020: Table 3).

**Table 3:** Changes in the retinal thicknesses in eyes with ERM.

Quadrant	Week 0	Week 2	Week 4	Week 8	P value
CFT (µm)	406.3 ± 79.9	403.2 ± 74.8	376.4 ± 65.6	355.9 ± 64.4	0.020
**INL (µm)**					
Temporal	70.9 ± 22.8	83.4 ± 29.8	77.4 ± 25.4	61.1 ± 19.2	0.003
Superior	91.8 ± 48.9	82.6 ± 27.3	76.1 ± 21.3	67.3 ± 22.7	0.184
Nasal	77.6 ± 27.5	91.8 ± 30.1	85.9 ± 25.8	77.8 ± 26.9	0.088
Inferior	69.5 ± 30.8	84.7 ± 22.5	72.4 ± 17.3	66.2 ± 20.3	0.137
**ORL (µm)**					
Temporal	238.9 ± 36.3	220.1 ± 28.5	218.9 ± 24.9	216.8 ± 24.3	0.065
Superior	221.6 ± 25.5	204.5 ± 23.7	205.9 ± 23.2	203.9 ± 21.7	0.039
Nasal	226.6 ± 28.1	235.7 ± 37.5	225.5 ± 28.9	216.3 ± 33.7	0.088
Inferior	235.8 ± 33.2	210.9 ± 23.1	208.1 ± 18.2	208.5 ± 15.2	0.006

Values are presented as means ± standard deviations.

*CFT* central foveal thickness, *INL* inner nuclear layer, *ORL* outer retinal layer (outer retinal layer + outer plexiform layer).

The mean preoperative INL thickness was 70.9 ± 22.8 µm in the temporal, 91.8 ± 48.9 µm in the superior, 77.6 ± 27.5 in the nasal, and 69.5 ± 30.8 µm in the inferior quadrants. The mean postoperative INL thickness was significantly thinner only in the temporal quadrant during follow-up period (*P* < 0.001).

The mean preoperative ORL thickness was 238.9 ± 36.3 µm in the temporal, 221.6 ± 25.5 µm in the superior, 226.6 ± 28.1 µm in the nasal, and 235.8 ± 33.2 µm in the inferior quadrant. The mean postoperative ORL thickness was significantly thinner only in the temporal quadrant during the follow-up period (*P* < 0.001).

### Relationships between displacements of retinal bifurcations and other factors

**Figure 4:**
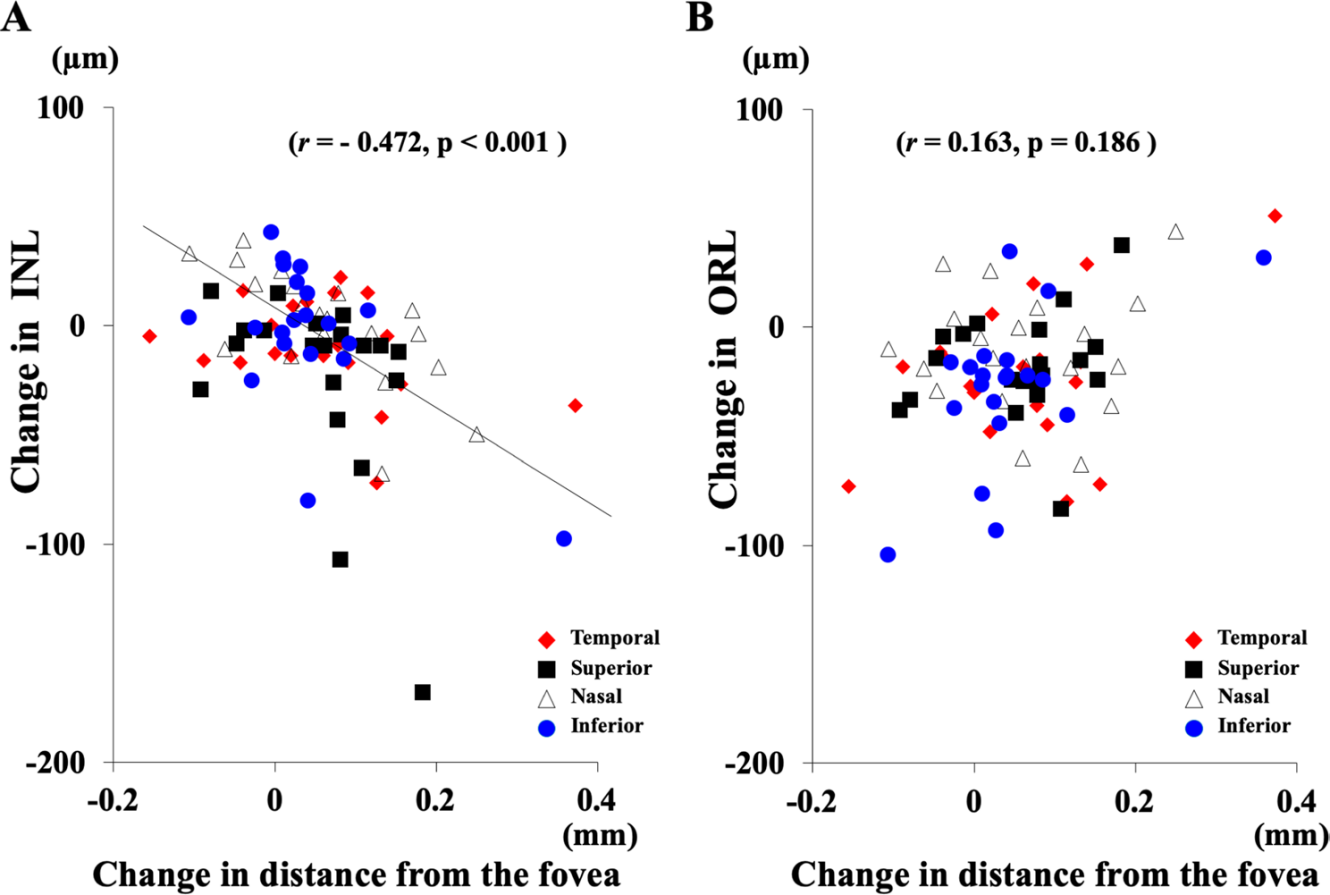
Graphs showing the relationship between the changes in the INL thickness and the changes in the distance from the bifurcations. The changes in the INL thickness were significantly correlated with the changes in the distance between the fovea for all the bifurcations in each eye (**A**), but there was no significant correlation in the change between the ORL thickness and the distance (**B**).

**Figure 5:**
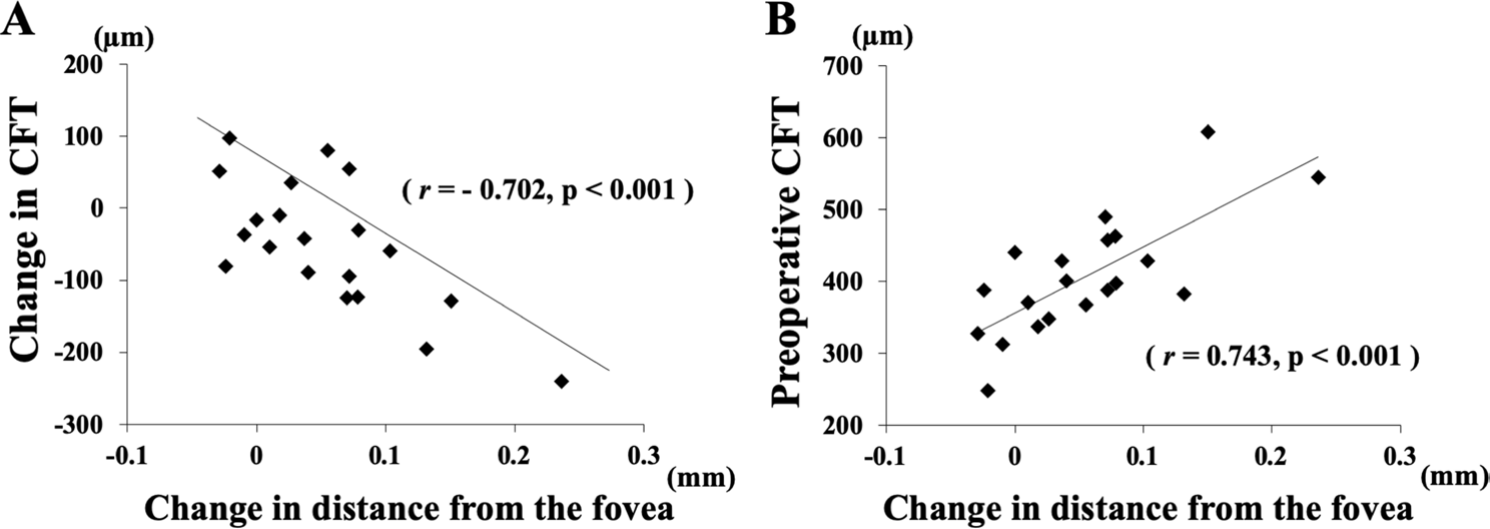
Correlation between the CFT and the distance from the fovea to the bifurcations. The change in CFT was significantly correlated with the change in the distance between the fovea and all the bifurcations (**A**). In addition, the preoperative CFT was significantly correlated with the change in the distance between the fovea and all of bifurcations (**B**).

**Figure 6:**
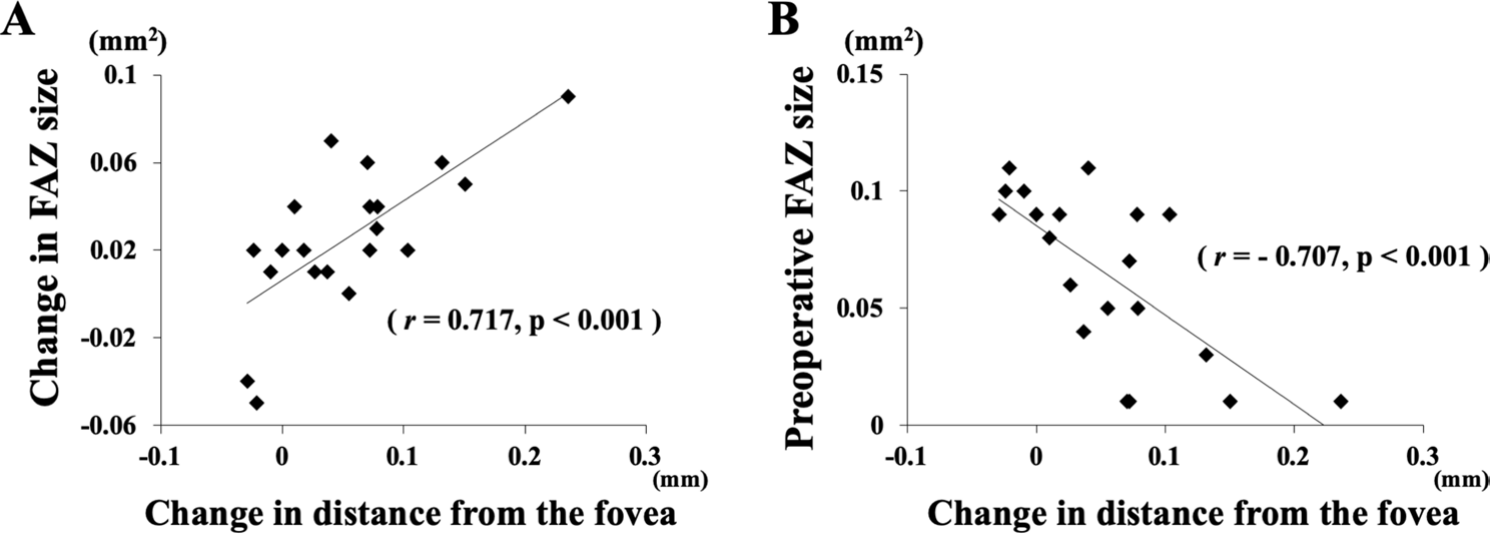
Correlation between the FAZ and the changes in the distance from the fovea to the bifurcations. The changes in FAZ area was significantly correlated with the change in the distance between the fovea and all of the bifurcations (**A**). In addition, the preoperative FAZ area was significantly correlated with the changes in distance between the fovea and all of the bifurcations (**B**).

Single linear regression analysis showed that the changes in the distance between the retinal bifurcations and the fovea was significantly correlated with the changes in the area of the FAZ (r = 0.717, *P* < 0.001; Fig. 4), the CFT (r = − 0.702, *P* < 0.001) (Fig. 5), and the INL thickness (r = − 0.702, *P* < 0.001; Fig. 6), but not significantly correlated with the pre-and postoperative BCVA. The degree of the displacement of each vessel bifurcation at any time point was not significantly correlated with its preoperative distance from the fovea. The ORL thickness was not significantly correlated with other variables, e.g. the retinal displacement, the area of FAZ, the CFT, and the INL thickness.

## Discussion

One of the advantages of using the OCTA images to evaluate the displacement is the ability to measure many points in a short time. We measured an average of 71.0 points for each eye in the OCTA images, which was a much larger number than previous reports^17–19^. Our results showed that the distance between the retinal bifurcations and the fovea was significantly displaced centrifugally and asymmetrically in all 4 quadrants after the removal of the ERM by vitrectomy with ILM peeling. The FAZ area became significantly smaller and the CFT significantly thicker than that of the fellow eyes before surgery, and the FAZ area was enlarged and the CFT became thinner after the surgery. The changes in the displacement of the retinal bifurcations was significantly correlated with that in the area of the FAZ, the CFT, and the INL thickness.

Recent advances in vitrectomy surgery and ERM removal with ILM peeling have improved surgical safety and visual outcomes for ERM patients. Actually, there have been reports showing that vitrectomy with ILM peeling resulted in better visual improvement and lower ERM recurrence rates^6,20^. Thus, we planted to perform ILM in addition to ERM peeling for eyes with ERM.

The retina was centrifugally displaced in all of the 4 quadrants, and the size of FAZ, which was much smaller than the fellow eye before surgery and was enlarged with time after the surgery. These results clearly show that the retina in the macular region was significantly displaced centrifugally after the surgery and which is consistent with previous reports^17–19^.

In comparing the displacements between the inner and outer sectors of the superior and inferior quadrants, the distance in those sectors should be similar from the optic disc and the nasal displacement caused by the ILM peeling should not be different. The displacement of the bifurcation in the outer sector was greater than that in the inner sector in those quadrants. Accordingly, the farther the bifurcations were to the fovea, the greater were the displacements. These results corroborate the centrifugal displacements after the surgery.

On the other hand, the displacements in the inner sectors were greater than that in the outer sectors only in the nasal quadrant. In addition, the retina in the nasal quadrant was displaced the most among the four quadrants after the surgery. It has been reported that the retina is displaced nasally after ILM peeling in eyes with retinochoroidal diseases^21,22^ including eyes with a macular hole^16,23,24^. Consequently, the retina in the macula area was displaced nasally in addition to centrifugally in our cohort. Akahori et al. reported that there was greater displacements of the bifurcations farther from the optic disc^14^. The postoperative asymmetrical displacement in the temporal and nasal quadrants can be explained as a displacement of the nasal retina by the same directional forces, viz., centrifugally and nasally, but the temporal retina was displaced by different directional forces. This asymmetrical displacement might affect the recovery of the visual dysfunction and distortion postoperatively.

Our study showed that the INL thickness became thinner after surgery and the change in the INL thickness was significantly correlated with the degree of retinal displacement. These results imply that the INL is thickened by a centripetal traction of the ERM on the macula resulting in the development of aniseikonia and metamorphopsia. The release of the traction causes the retinal displacement which is correlated with the reduction of the INL thickness. These observations support the earlier findings^25–28^.

A contraction of the ERM exerts tangential traction on the inner retina and overlying vessels, causing a movement of the retinal vessels toward the center^29,30^. In addition, the preoperative FAZ area and the CFT were correlated with the displacement of retina. Furthermore, the change in the FAZ area was correlated with the displacement of the bifurcations and the change in the CFT which is in good agreement with a previous report. These results suggest that the enlarged FAZ is related to the greater displacement of retina and reduction of the CFT. There have been several reports showing an association of the displacement of the retina with the changes in the retinal structures using OCT^31^ or retinal vasculature using OCTA^32,33^. Our results corroborate these earlier reports. Thus, a smaller preoperative FAZ and a thicker CFT may indicate greater macular contraction caused by the traction of the ERM and the progression of the disease process. Taken together, these findings indicate that the horizontal contraction in the macular area is correlated with the vertical contraction of the retina in eyes with an ERM.

In our cohort, the BCVA was not significantly correlated with the retinal displacement. There are several explanations for this. First, we evaluated the inner retinal displacement because retinal vessels are located in the inner retina but the structure of the outer retina may be more important for vision than the retinal displacements^34^. Second, there are several reports that the structure at the fovea is related to the vision in many retinochoroidal diseases which was not evaluated^11,35^.

This study has several limitations. First, we evaluated the displacement after a relatively short period of 8 weeks after the surgery. Second, the mean size of the ERM and ILM peeled was 4.78 ± 0.44 DD, and the size was not consistent among the eyes. Third, an imaging of the 6 × 6 mm area would be more helpful in terms of recognizing vasculature in a wider area. However, the resolution of the image is worse than that in smaller area of 3 × 3 mm, and this would make it more difficult to detect the bifurcations. Thus, we chose to use the 3 × 3 mm image. Fourth, we did not evaluate the eyes for aniseikonia and metamorphopsia, and it might have been better to investigate these parameters in addition to the BCVA to determine the functional changes after surgery. To resolve these limitations, prospective studies on a larger number of eyes with a longer follow-up periods are needed.

In conclusion, the distance between the retinal bifurcations and the fovea was asymmetrically increased after removal of the ERM by vitrectomy with ILM peeling. The change in the distances was correlated with the changes in the FAZ area and the CFT. These results indicate that a horizontal macular contraction is correlated with vertical retinal contraction in the eyes with an ERM.
